# Reframing Mother–Child Closeness and Cooperative Caregiving: A Comparative Study of Two Hunter‐Gatherer Populations

**DOI:** 10.1002/ajpa.70345

**Published:** 2026-08-14

**Authors:** Ayana Tanaka, Haneul Jang, Shun Hongo, Akira Takada

**Affiliations:** ^1^ Graduate School of Human‐Environment Studies Kyushu University Fukuoka Japan; ^2^ Japan Society for the Promotion of Science Tokyo Japan; ^3^ UMI Source Université Paris‐Saclay, UVSQ, IRD Guyancourt France; ^4^ Research Institute for Humanity and Nature Kyoto Japan; ^5^ The Hakubi Center for Advanced Research Kyoto University Kyoto Japan; ^6^ Graduate School of Asian and African Area Studies Kyoto University Kyoto Japan

**Keywords:** Africa, attachment, comparison of populations, cooperative childcare, hunter‐gatherers

## Abstract

**Objectives:**

Caregiving during early childhood emerges from a dynamic interplay between maternal and allomaternal contributions. This study aimed to challenge polarized portrayals of hunter‐gatherer childcare as either “mother‐centered” or “cooperative” and provide empirical evidence for an integrative cross‐cultural approach.

**Materials and Methods:**

We compared caregiving structures in two hunter‐gatherer populations positioned at contrasting ends of a continuum of child‐rearing strategies: the !Xun in southern Africa, portrayed as emphasizing mother–child closeness, and the Baka in central Africa, described as exemplifying cooperative caregiving. Over 500 h of caregiving interactions involving 40 children (1–59 months) were recorded using a standardized focal‐follow protocol with 30‐s interval coding. Rates of maternal, allomaternal, and peer‐based caregiving behaviors were analyzed using generalized additive mixed models.

**Results:**

Maternal holding did not differ between the !Xun and the Baka, underscoring the central role of mothers during infancy. However, breastfeeding was less frequent among the !Xun than among the Baka. Allomaternal holding was less frequent among the !Xun than among the Baka. When focusing on child and adolescent allomaternal caregivers, child‐holding was more frequent among the !Xun than among the Baka during a period that included the likely !Xun weaning period. Care by young allomaternal caregivers may have played a supportive role during the earlier weaning period associated with sedentarization.

**Discussion:**

Human caregiving systems should not be understood as either “mother‐centered” or “cooperative”. Rather, they should be understood as flexible combinations of interrelated components—including maternal, allomaternal, and peer‐based care—shaped by ecological, social, and developmental factors.

## 1 Introduction

Across human societies, caregiving during early childhood emerges from a dynamic interplay between maternal and nonmaternal (or allomaternal) contributions (Hewlett and Winn 2014; Hrdy 1999, 2009; Kramer 2005, 2010). This diversity in caregiving arrangements has long been of interest to anthropology, psychology, and developmental science because it provides insight into how human life history evolved and how flexibly human caregiving systems can respond to different environmental conditions. Alongside mother–child closeness and mothers' typical role as primary caregivers, substantial support from other group members, such as fathers, grandparents, siblings, and peers, has been widely documented and is often referred to as cooperative caregiving or alloparenting (Chaudhary, Page, et al. 2024; Emmott and Page 2019; Hawkes et al. 1998; Helfrecht et al. 2020; Hrdy 1999, 2009; Kramer 2005, 2010). However, important questions remain about how caregiving roles are distributed and to what extent maternal care remains central across diverse ecological and social contexts.

We address these questions through a comparative analysis of early childhood caregiving in two hunter‐gatherer populations: the settled !Xun of the semiarid desert of Namibia and the semisettled Baka of the tropical rainforests of Cameroon. These groups have historically been portrayed as occupying contrasting positions along a continuum of child‐rearing strategies. The !Xun, hunter‐gatherers of southern Africa like the Juǀ'hoan (!Kung), are often cited as examples of intensive maternal care, characterized by frequent mother–child physical contact (Bowlby 1969; Draper 1976; Konner 1977; Takada 2020). In contrast, the Baka, the hunter‐gatherers of central Africa like the Aka and Efe, are emphasized for their extensive allomaternal involvement, with childcare frequently shared among fathers, siblings, grandparents, and other community members (Chaudhary, Salali, et al. 2024; Hewlett 1991; Hirasawa 2005; Meehan and Hawks 2013; Meehan et al. 2014; Tronick et al. 1987). These portrayals have shaped broader debates in evolutionary anthropology, with Juǀ'hoan caregiving often cited in support of attachment theora (Bowlby 1969; Konner 1977) and Aka and Efe caregiving emphasized in the development of cooperative childcare models (Hewlett 1991; Hrdy 1999, 2009).

Although these two hunter‐gatherer populations have often been portrayed in contrasting ways, previous research suggests that this contrast is not clear‐cut. Even in societies characterized by intensive maternal care, caregiving occurs within broader kinship networks that provide additional childcare support (Konner 2017; Kruger and Konner 2010; Takada 2014). Conversely, in societies characterized by extensive allomaternal involvement, mothers continue to provide the majority of care and remain children's primary attachment figures (Meehan and Hawks 2013). These observations raise two important questions: to what extent does the apparent contrast between intensive maternal care and extensive allomaternal involvement reflect real variation in caregiving practices, and to what extent is it shaped by methodological inconsistencies? Despite decades of research on hunter‐gatherer childcare, fundamental quantitative descriptions of cross‐cultural variation in maternal and allomaternal care remain limited because previous studies have differed in their observational methods, sample age ranges, and analytical frameworks, making direct comparison difficult.

This study aims to challenge polarized portrayals of hunter‐gatherer childcare as either “mother‐centered” or “cooperative” and to provide empirical evidence for an integrative cross‐cultural approach. To address this aim, we employed a standardized focal‐follow protocol to directly compare early childhood caregiving between the !Xun in Namibia and the Baka in Cameroon, two populations that have often been positioned at contrasting ends of a continuum of child‐rearing strategies. For the !Xun, we used data previously collected and reported by Takada (2010, 2014). Takada's studies considered !Xun caregiving in relation to published observations of the Juǀ'hoan, another southern African hunter‐gatherer population, and found broadly consistent patterns of mother–child closeness across the two populations. At the same time, compared with the Juǀ'hoan, the !Xun showed earlier weaning, greater involvement of older children in caring for younger children, and earlier participation in mixed‐age peer groups. These differences were interpreted as possible effects of sedentarization. Building on these findings, the present study combined the previously collected !Xun dataset with newly collected Baka data, yielding more than 500 h of focal observations of 40 children aged 1–59 months. The use of a common observational and analytical framework helps distinguish variation in caregiving practices from methodological inconsistencies and enables us to examine both shared and divergent caregiving patterns.

In this study, we compared maternal and allomaternal caregiving—specifically, child‐holding and breastfeeding—with particular attention to interactions involving young allomaternal caregivers, including child‐holding and focal children's participation in mixed‐age peer groups. We included participation in mixed‐age peer groups because older children and adolescents in these groups often supervise and care for toddlers (Page et al. 2021). Such groups therefore offer insights into how caregiving responsibilities and competencies are distributed beyond adult–child dyads. Because these measures may vary with child age as children are weaned, become independently mobile, and participate increasingly in social activities, we examined age‐related variation in our comparison of the !Xun and Baka. By comparing these dimensions between the !Xun and the Baka within the same methodological framework, our study contributes empirical evidence to a growing body of research in evolutionary and biological anthropology that moves beyond classifying human caregiving as either “mother‐centered” or “cooperative” toward a more nuanced understanding of caregiving systems shaped by ecological, social, and developmental factors.

## 2 Materials and Methods

### 2.1 Ethics Statement

This research was conducted as part of the research project “Ecological future making of child‐rearing in contact zones between hunter‐gatherers and agro‐pastoralists in Africa” (Research representative: Akira Takada) and received ethical approval from the Ethical Review Committee of the African Center for Area Studies (Review Numbers: 22‐10B; 22‐05C). Takada conducted research in Namibia with research permission obtained from the Namibian government. Tanaka conducted research in Cameroon under an agreement for a joint intergovernmental SATREPS research project between Cameroon and Japan. Our study procedures and methods complied with national laws, relevant guidelines, and regulations of Namibia and Cameroon. Participants were children aged 0–4 years living in the study villages. After a detailed explanation of the study, including the focal observation procedures, written or oral informed consent was obtained from a parent. Participation was voluntary, and consent could be withdrawn at any time without penalty. All individual‐level information was anonymized. We consulted with community representatives before, during, and after the study and will disseminate the research findings to relevant stakeholders through community meetings.

### 2.2 Data Collection

The data analyzed in this study were obtained through focal observations in which an individual child was continuously followed and the child's behaviors and interactions with caregivers were recorded throughout the observation period. The !Xun data used in the present study were drawn from data previously collected by Takada during fieldwork conducted in August 2004 in a Namibian village where 143 !Xun individuals resided (Takada 2010, 2014). From these data, we analyzed selected behavioral measures relevant to the present study. The Baka dataset was newly collected by Tanaka from February to July 2023 in a Cameroonian village where 138 Baka individuals resided. Ethnographic descriptions, along with demographic data for the Baka and !Xun (Tables S1 and S2), are included in the Supporting Information. We did not include observations at overnight forest camps in the dataset. Takada recorded data for the !Xun using a pen on field data sheets, and Tanaka recorded data for the Baka using a smartphone with the CyberTracker application. All children aged 0–4 years in both villages—17 !Xun and 23 Baka—were studied (Table S3). The ages of !Xun children ranged from 5 to 59 months (determined by governmental birth records); the ages of Baka children ranged from 1 to 59 months (determined by interviews with their parents).

During each focal observation session, the observer continuously followed a single focal child and recorded behaviors involving that child, including interactions with caregivers and peers. The analyzed dataset comprised 136 h of focal‐follow data for the !Xun and 368 h for the Baka. For each focal child, the observation time was equivalent to one full day (8 h) for the !Xun and two full days (16 h) for the Baka, with observations distributed across several field days. Observation bouts were conducted within one of three daytime blocks (8:00–11:00 a.m., 11:00 a.m.–3:00 p.m., or 3:00–6:00 p.m.). Within each bout, we repeated a 48‐min recording session followed by a 12‐min break. Each recording session was divided into consecutive 30‐s intervals, and the target behaviors of children and caregivers were coded as present (1) if they occurred at least once in the interval or absent (0) otherwise. This design yielded 960 binary records per focal child per behavior for the !Xun and 1920 for the Baka (Table S4).

Using behavioral categories adapted from Takada (2010, 2014), we examined how caregiving behaviors varied with focal children's age and between the two populations. To evaluate the distinction between “mother‐centered” and “cooperative” childcare empirically, we examined (1) maternal caregiving behaviors, focusing on child‐holding and breastfeeding by mothers and (2) allomaternal caregiving behaviors, focusing on child‐holding and breastfeeding by allomaternal individuals, including adults and older children/adolescents. Additionally, we looked into young allomaternal caregivers (individuals who had not yet married) by examining (3) young allomaternal caregiver interactions with focal children. These interactions included child‐holding by young allomaternal caregivers as well as focal children's participation in mixed‐age peer groups. “Child‐holding” included cradling, carrying in a cloth sling, and back‐carrying. “Breastfeeding” was defined as any nipple‐suckling by the focal child, irrespective of milk transfer. In hunter‐gatherer societies, mixed‐age peer groups serve as important contexts in which children acquire and transmit subsistence skills, knowledge, and social norms through play (Gallois et al. 2018; Lew‐Levy and Amir 2026). We coded “participation in mixed‐age peer groups” as present when the focal child took part in the activity of a group of children and adolescents that did not include adults.

By the time of the focal observations, both researchers had been making repeated visits to their respective study villages for approximately 6 years. Children and caregivers were therefore accustomed to the researchers' presence, which likely helped reduce observer effects. Participation was voluntary, and no parents of eligible children declined participation. Focal observations were conducted with due consideration for the circumstances of eligible children, including whether they were present in the village rather than away for extended stays at forest camps. No focal observation sessions were interrupted or terminated prematurely.

### 2.3 Data Analyses

The statistical analysis was conducted using R version 4.5.0 (R Core Team 2025) and RStudio version 2023.09.1+494 (Posit Team 2023). To model the effects of age and population on the interval‐level probability of target behaviors, we used generalized additive mixed models (GAMMs) constructed with the R package “gamm4” version 0.2‐7 (Wood and Scheipl 2025). GAMMs extend generalized additive models (GAMs) by incorporating both nonlinear relationships and random effects. For this analysis, we adapted R code provided by Hongo et al. (2022).

We assumed a binomial distribution with a logit link function for the response variables. Five response variables were defined: (1a) rate of maternal child‐holding, (1b) rate of maternal breastfeeding, (2) rate of allomaternal child‐holding, (3a) rate of young allomaternal child‐holding, and (3b) rate of focal children's participation in mixed‐age peer groups. We calculated behavioral rates as the proportion of 30‐s intervals in which the behavior occurred at least once (coded 1), divided by the total number of intervals for each focal child. These behavioral rates served as response variables in three GAMMs designed to assess how caregiving behaviors varied between the two groups. Model 1 (age‐only model) included only a smooth term for focal child's age to account for age‐dependent variation in caregiving behaviors. In Model 2 (age + population model), we added a parametric term for population (!Xun vs. Baka) to examine whether there were overall differences in the rate of caregiving behaviors between populations. Model 3 (age × population model) further included an interaction term between age and population to examine whether there were differences in the age‐related patterns of caregiving behaviors between the populations. We also included random intercept effects of focal child ID and observation ID in all candidate models to account for potential variations among individuals and observation dates.

To describe the data, we first summarized the observed behavioral rates for each population and age category. We then compared the three candidate models using likelihood ratio tests. The more complex model was selected when it significantly improved model fit (Model 2 over Model 1, and Model 3 over Model 2); otherwise, the simpler model was retained. Model‐predicted trajectories from the selected model were used to describe age‐related trends in caregiving behaviors and, where applicable, differences between populations across the observed age range. Statistical significance was set at *p* < 0.05.

## 3 Results

### 3.1 Comparison of Maternal Caregiving Behaviors Between !Xun and Baka

#### 3.1.1 Maternal Child‐Holding

Overall, maternal child‐holding was observed at similar rates in the !Xun and the Baka, occurring in 16% of observed intervals among the !Xun and 17% among the Baka across observations of children up to 59 months of age. Among the !Xun, the observed rate was 27% for children younger than 30 months and 1% for those aged 30–59 months. Among the Baka, the corresponding rates were 28% and 4%, respectively. In both populations, the observed rate of maternal child‐holding declined markedly from the younger to the older age category.

**FIGURE 1:**
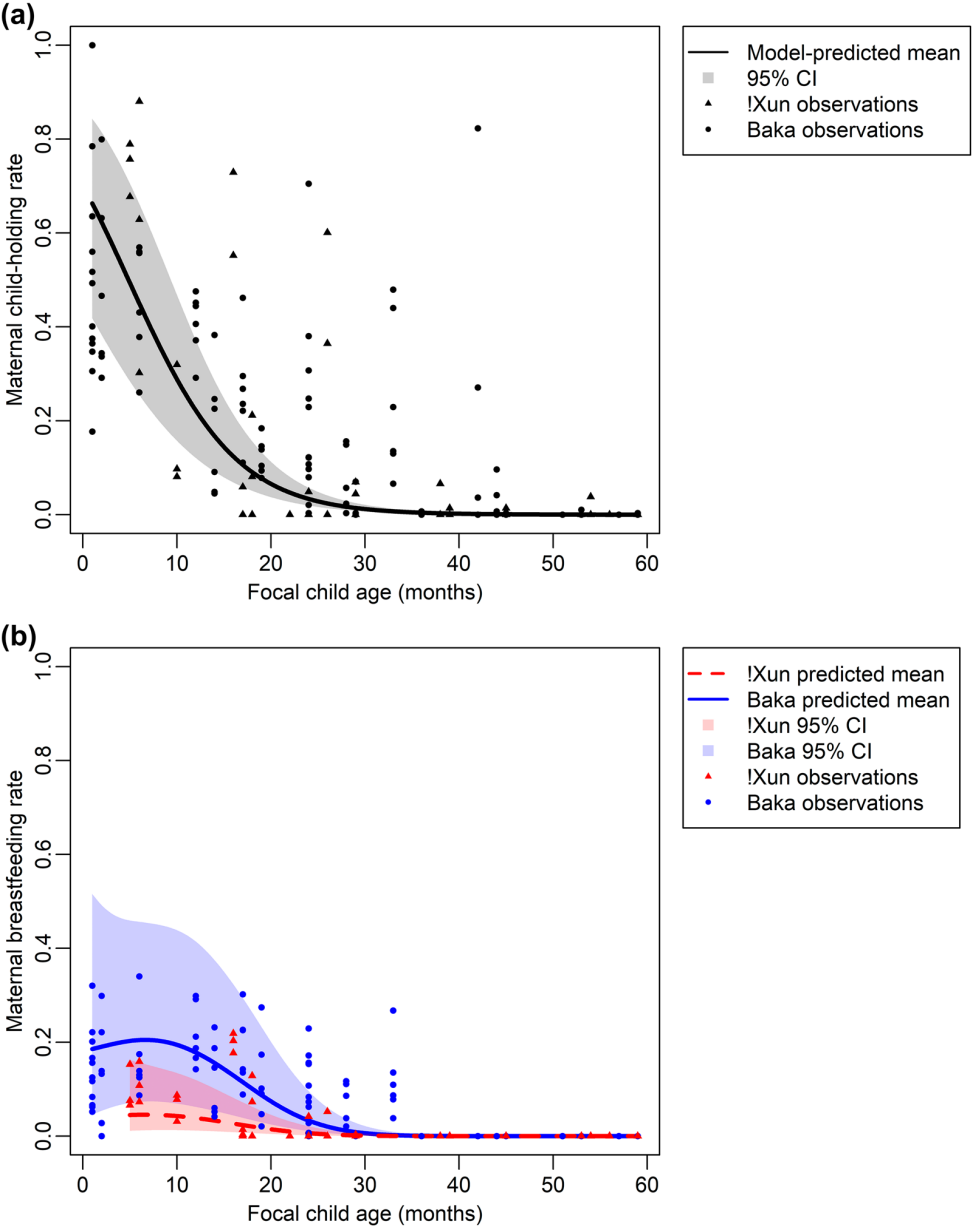
Age‐related variation in maternal caregiving behaviors among the !Xun in Namibia and the Baka in Cameroon. (a) Maternal child‐holding declined with focal child age in both populations, with no evidence of a population difference. Triangles represent the !Xun; circles represent the Baka. A single line indicates the model‐predicted trend shared by both populations based on the selected model; the shaded area denotes the 95% confidence interval. (b) Maternal breastfeeding declined with focal child age in both populations, with higher model‐predicted rates among the Baka. Triangles and dotted lines represent the !Xun; circles and solid lines represent the Baka. Lines indicate model‐predicted trends based on the selected model; shaded areas denote 95% confidence intervals.

Likelihood ratio tests identified the age‐only model (Model 1) as the selected model (Model 1 vs. Model 2: χ² = 1.42, *p* = 0.234; Model 2 vs. Model 3: χ² = 0.003, *p* = 0.999; Table S5), providing no evidence of an overall difference in maternal child‐holding between the !Xun and the Baka. The shared model‐predicted trajectory showed that maternal child‐holding declined rapidly with age, reaching approximately 1% by around 30 months and declining further toward zero thereafter (Figure 1a). This decline coincided with weaning and children's increasing independence in locomotion and feeding.

#### 3.1.2 Maternal Breastfeeding

Overall, maternal breastfeeding was observed at lower rates in the !Xun than in the Baka, occurring in 3% of observed intervals among the !Xun and 7% among the Baka across observations of children up to 59 months of age. Among the !Xun, the observed rate decreased from 6% among children younger than 30 months to 0% among those aged 30–59 months. Among the Baka, the corresponding rates were 13% and 1%, respectively. In both populations, the observed rate of maternal breastfeeding declined markedly from the younger to the older age category. The youngest !Xun child for whom no breastfeeding was recorded was 22 months old, whereas the oldest child observed being breastfed was 29 months old. Among the Baka, the corresponding ages were 29 and 33 months, respectively.

Likelihood ratio tests identified the age + population model (Model 2) as the selected model (Model 1 vs. Model 2: χ² = 4.69, *p* = 0.030; Model 2 vs. Model 3: χ² = 1.72, *p* = 0.423; Table S6), indicating an overall difference in maternal breastfeeding between the !Xun and the Baka but similar age‐related trajectories. In both populations, the predicted breastfeeding rate peaked at around 7 months and then declined rapidly. Maternal breastfeeding was consistently more frequent among the Baka than among the !Xun across the modeled age range, with no crossing of the fitted curves (Figure 1b). At 24 months, the predicted breastfeeding rate was 0.6% (95% CI: 0.2%–2.1%) among the !Xun and 3.3% (95% CI: 1.1%–9.6%) among the Baka. At 30 months, the predicted breastfeeding rate was approximately 0.1% (95% CI: 0.03%–0.5%) among the !Xun and 0.6% (95% CI: 0.2%–2.1%) among the Baka. Taken together, the observed ages and model‐based patterns suggest that weaning typically occurred at around 2–2.5 years of age among the !Xun and around 2.5–3 years of age among the Baka.

### 3.2 Comparison of Allomaternal Caregiving Behaviors Between !Xun and Baka

#### 3.2.1 Allomaternal Child‐Holding

Overall, allomaternal child‐holding was observed at lower rates in the !Xun than in the Baka, occurring in 9% of observed intervals among the !Xun and 15% among the Baka across observations of children up to 59 months of age. Among the !Xun, the observed rate was 15% for children younger than 30 months and 1% for those aged 30–59 months. Among the Baka, the corresponding rates were 24% and 5%, respectively. In both populations, the observed rate of allomaternal child‐holding declined markedly from the younger to the older age category.

**FIGURE 2:**
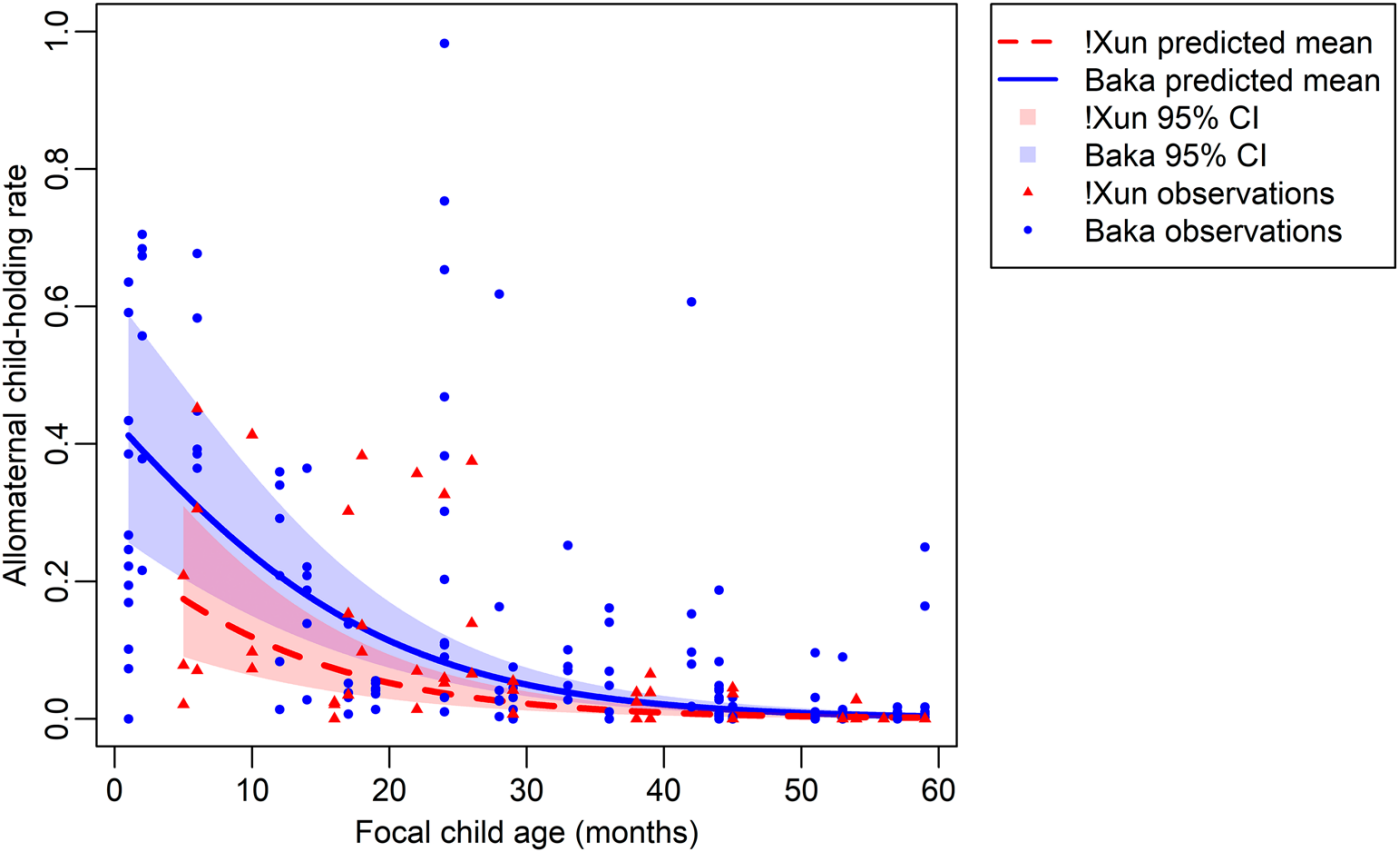
Age‐related variation in allomaternal caregiving behavior among the !Xun in Namibia and the Baka in Cameroon. Allomaternal child‐holding declined with focal child age in both populations, with higher model‐predicted rates among the Baka. Triangles and dotted lines represent the !Xun; circles and solid lines represent the Baka. Lines indicate model‐predicted trends based on the selected model; shaded areas denote 95% confidence intervals.

Likelihood ratio tests identified the age + population model (Model 2) as the selected model (Model 1 vs. Model 2: χ² = 4.82, *p* = 0.028; Model 2 vs. Model 3: χ² = 3.35, *p* = 0.187; Table S7), indicating an overall difference in allomaternal child‐holding between the !Xun and the Baka but similar age‐related trajectories. In both populations, the predicted rate of allomaternal child‐holding declined monotonically with age. It remained consistently higher among the Baka than among the !Xun across the modeled age range, with no crossing of the fitted curves (Figure 2).

#### 3.2.2 Allomaternal Breastfeeding

No allomaternal breastfeeding was observed among the !Xun during these focal observations. In contrast, allomaternal breastfeeding was observed among the Baka, involving infants aged 1, 2, and 6 months. The allomaternal caregivers included the focal child's maternal aunt, maternal grandmother, paternal grandmother, and a nonkin caregiver (the wife of the paternal grandfather's younger brother). During these focal observations, no instances of breastfeeding by allomaternal caregivers were observed among Baka children over 6 months of age. Therefore, statistical analysis was not conducted. Allomaternal breastfeeding during infancy is relatively common among central African hunter‐gatherers (Hewlett and Winn 2014), but not among southern African hunter‐gatherers. Among the !Xun, allomaternal breastfeeding is customarily not permitted (Takada 2020). The differences in allomaternal breastfeeding between the !Xun and the Baka may reflect differences in cultural norms between the two societies.

### 3.3 Comparison of Young Allomaternal Caregiver Interactions With Focal Children Between !Xun and Baka

#### 3.3.1 Young Allomaternal Child‐Holding

Overall, child‐holding by young allomaternal caregivers was observed at similar rates in the !Xun and the Baka, occurring in 8% of observed intervals among the !Xun and 9% among the Baka across observations of children up to 59 months of age. Among the !Xun, the observed rate was 13% for children younger than 30 months and 1% for those aged 30–59 months. Among the Baka, the corresponding rates were 15% and 3%, respectively. In both populations, the observed rate of child‐holding by young allomaternal caregivers declined markedly from the younger to the older age category.

**FIGURE 3:**
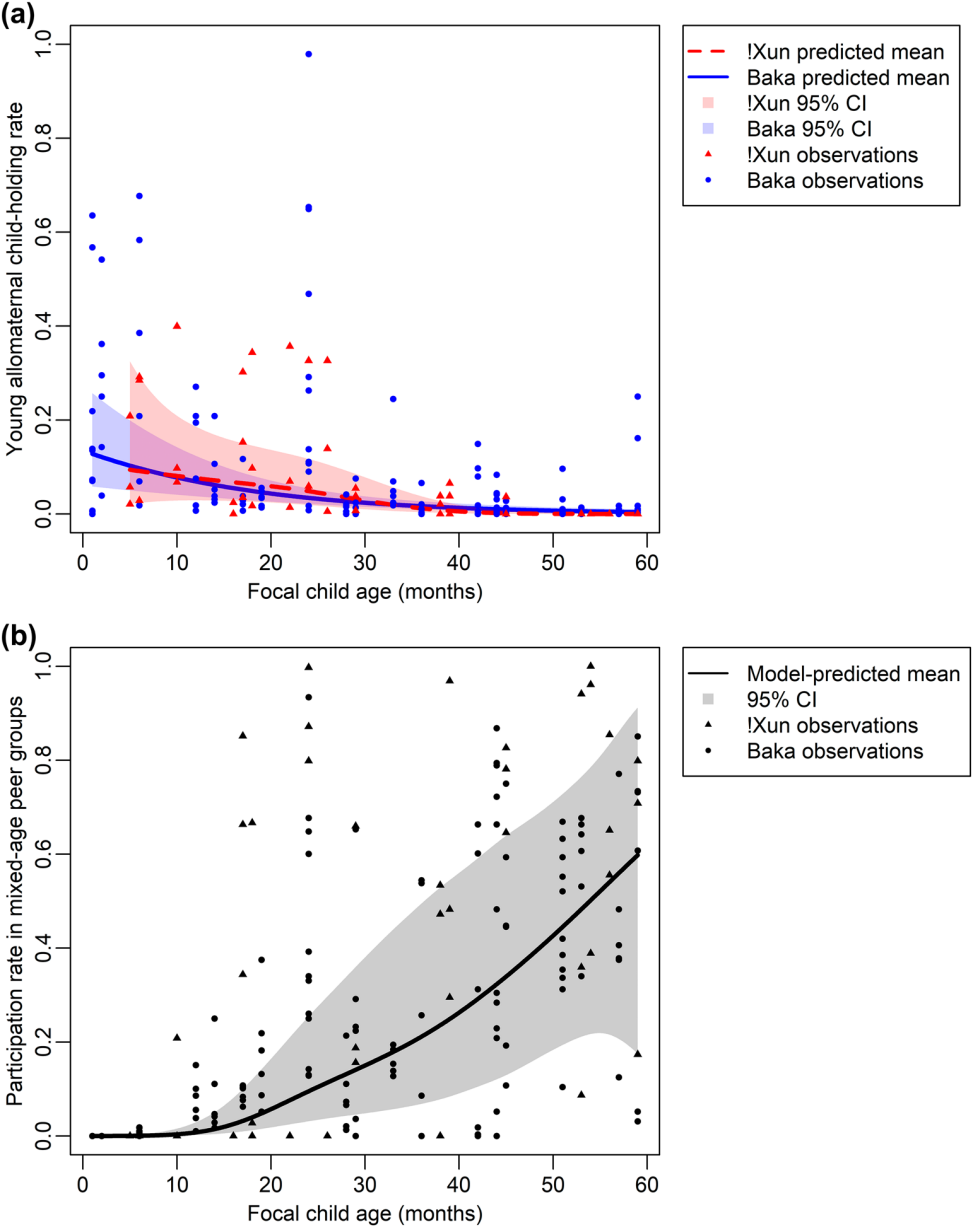
Age‐related variation in young allomaternal caregiver interactions among the !Xun in Namibia and the Baka in Cameroon. (a) Young allomaternal child‐holding declined with focal child age in both populations, with distinct model‐predicted trajectories in the two populations. Triangles and dotted lines represent the !Xun; circles and solid lines represent the Baka. Lines indicate model‐predicted trends based on the selected model; shaded areas denote 95% confidence intervals. (b) Focal children's participation in mixed‐age peer groups increased with focal child age in both populations, with no evidence of a population difference. Triangles represent the !Xun; circles represent the Baka. A single line indicates the model‐predicted trend shared by both populations based on the selected model; the shaded area denotes the 95% confidence interval.

Likelihood ratio tests identified the age × population model (Model 3) as the selected model (Model 1 vs. Model 2: χ² = 1.64, *p* = 0.200; Model 2 vs. Model 3: χ² = 9.92, *p* = 0.007; Table S8), indicating that population differences in young allomaternal child‐holding varied with focal child age. In both populations, the predicted rate of child‐holding by young allomaternal caregivers declined monotonically with age. The fitted curves crossed twice: the predicted rate was slightly higher among the Baka up to around 8 months of age, higher among the !Xun from around 8 to 33 months, and higher again among the Baka after around 33 months (Figure 3a).

#### 3.3.2 Focal Children's Participation in Mixed‐Age Peer Groups

Among the !Xun, the observed rates were 3% for children younger than 12 months, 30% for those aged 12–29 months, and 61% for those aged 30–59 months. Among the Baka, the corresponding rates were less than 1%, 19%, and 41%, respectively. In both populations, the observed rate of participation in mixed‐age peer groups increased with age.

Likelihood ratio tests identified the age‐only model (Model 1) as the selected model (Model 1 vs. Model 2: χ² = 0.20, *p* = 0.652; Model 2 vs. Model 3: χ² = 0.92, *p* = 0.632; Table S9), providing no evidence of an overall difference in focal children's participation in mixed‐age peer groups between the !Xun and the Baka. The shared model‐predicted trajectory showed that participation increased steadily with age, remaining below 1% through 12 months and then increasing more markedly around the time when independent locomotion typically emerges, reaching approximately 60% at 59 months (Figure 3b).

## 4 Discussion

This study aimed to challenge polarized portrayals of hunter‐gatherer childcare as either “mother‐centered” or “cooperative” and to provide empirical evidence for an integrative cross‐cultural approach. To this end, we compared the !Xun in southern Africa and the Baka in central Africa, whose childcare practices have traditionally been described as “mother‐centered” and “cooperative”, respectively. Using standardized observational methods across both populations, we directly assessed similarities and differences between the !Xun and the Baka in maternal, allomaternal, and peer‐based care. Detailed descriptions of !Xun infants, young children, and their caregivers are available in Takada (2010, 2014); here, we focus on a standardized comparison of the previously collected !Xun data with newly collected Baka data.

Maternal care followed a broadly similar trajectory in both the !Xun and the Baka. For both groups, maternal holding was high during infancy and declined steeply with age (Figure 1a). This is consistent with the age‐related decline in breastfeeding (Figure 1b) along with toddlers' increasing body mass and mobility, and highlights the central role of mothers in infancy. The primary difference in maternal care between the !Xun and the Baka lies in maternal breastfeeding. Maternal breastfeeding was less frequent among !Xun children than among Baka children. Weaning timing differed between the two populations: weaning typically occurred around 2–2.5 years of age among the !Xun, compared with around 2.5–3 years of age among the Baka. This earlier weaning pattern among the !Xun, relative to the Baka, is likely influenced by sedentarization, particularly through the availability of weaning foods such as porridge, cattle milk, and soft drinks made from pearl millet. By comparison, among the Ju/’hoan, another nomadic southern African hunter‐gatherer group, children were typically not weaned until 3–4 years of age (Draper 1976; Konner 1977). Taken together, these findings support the central role of mothers during infancy across diverse ecological and social contexts, while also showing that—even in settings where closeness to the mother is highly valued—maternal breastfeeding practices vary with ecological and social conditions.

Turning to allomaternal care, the level of allomaternal holding was higher among the Baka than among the !Xun (Figure 2). This difference likely reflects maternal energetic trade‐offs associated with Baka subsistence activities, which are physically demanding and often require mothers to leave infants with other caregivers or to pass them among group members. For example, tasks such as digging wild yams, fishing, or harvesting plantains limit the possibility of carrying children throughout the activity. In such contexts, Baka mothers frequently leave their children with other caregivers in the village or pass their infants to others during subsistence trips, similar to practices reported among the BaYaka hunter‐gatherers in the Republic of the Congo (Jang et al. 2022; Visine et al. 2024). In contrast, !Xun mothers rarely leave their infants with others while engaged in daily subsistence activities (Takada 2014, 2020). Their primary tasks—such as planting and harvesting pearl millet or gathering wild fruits and nuts—are generally less labor‐intensive, often permitting mothers to back‐carry their children while working (Takada 2022). This perspective highlights how caregiving systems are shaped by ecological factors.

Finally, patterns of care provided by young allomaternal caregivers (older children and adolescents) further illustrate the flexibility of caregiving systems in relation to ecological, social, and developmental factors. Child‐holding by young allomaternal caregivers declined with age in both populations, but the age‐related patterns differed between the !Xun and the Baka (Figure 3a). Among the !Xun, young allomaternal child‐holding was more common than among the Baka from around 8 to 33 months, an age range that included the likely weaning period for !Xun children (approximately 2–2.5 years of age). This temporal overlap suggests that care provided by older children and adolescents may have played a supportive role during the weaning period in the context of sedentarization and access to weaning foods. Regarding focal children's participation in mixed‐age peer groups, it increased with age in both the !Xun and the Baka (Figure 3b). Such groups may act as “collective allomothers,” combining caregiving, play, and cultural learning (Gallois et al. 2018; Lew‐Levy and Amir 2026; Page et al. 2021), and supplementing maternal care as it declines. These findings suggest that the !Xun caregiving system, although traditionally characterized as “mother‐centered,” also has aspects that can be considered “cooperative”.

Taken together, these findings indicate that maternal care and allomaternal care should not be construed as mutually exclusive, nor should human caregiving systems be framed as either “mother‐centered” or “cooperative”. Rather, maternal, allomaternal, and peer‐based care should be understood as interrelated components of caregiving systems shaped by ecological, social, and developmental factors. These components are combined in flexible ways within each caregiving system. By applying a consistent methodology across the two populations, which have traditionally been associated with opposing models, this study provides empirical support for a more integrative account of human caregiving. This integrative perspective on human caregiving reframes long‐standing debates in anthropology, psychology, and human developmental science.

The substantial presence of allomaternal caregiving, at least in the two populations examined here, is consistent with evolutionary perspectives that emphasize cooperative breeding in humans (Hrdy 1999, 2009). Maternal predominance in early infancy does not diminish the importance of care provided by others, nor does allomaternal involvement undermine the mother–child relationship. At the same time, breastfeeding practices, allomaternal caregiving, and caregiving by adolescents and older children vary across ecological and social contexts, including those characterized by subsistence practices and levels of sedentarization (Page et al. 2023), as well as across stages of child development. Expanding cross‐population comparisons to include additional populations will help determine which features of caregiving are widely shared and which vary with specific ecological, social, and developmental conditions. In particular, examining how components of caregiving, including maternal, allomaternal, and peer‐based care, are combined within each population and comparing these configurations across populations will help clarify how variation in human caregiving practices fits within existing evolutionary frameworks. Viewing these configurations from an integrative perspective has implications for understanding cooperative childcare, developmental plasticity, and the evolution of human caregiving.

## Author Contributions

**Ayana Tanaka:** conceptualization, methodology, investigation (Baka), formal analysis, visualization, writing – original draft, and writing – review and editing. **Haneul Jang:** methodology, support for formal analysis, and writing – review and editing. **Shun Hongo:** support for formal analysis and writing – review and editing (including feedback on figures). **Akira Takada:** conceptualization, methodology, investigation (!Xun), supervision, funding acquisition, and writing – review and editing.

## Funding

The field research was funded by the “Ecological future making of child‐rearing in contact zones between hunter‐gatherers and agro‐pastoralists in Africa” project (principal investigator: Akira Takada; JP22H04929) and Coméca Project (principal investigator: Hirokazu Yasuoka; JPMJSA1702). Ayana Tanaka received funding from the Shibusawa Foundation for Ethnological Studies (fiscal year 2023). The open access publication charge was supported by JSPS KAKENHI (principal investigator: Ayana Tanaka; grant number: JP25K16477).

## Conflicts of Interest

The authors declare no conflicts of interest.

## Supporting information


**Table S1:** Age‐sex composition of !Xun residents of Ekoka (census by Takada in 1998).
**Table S2:** Age‐sex composition of Baka residents in Lanjo (census by Tanaka in 2023).
**Table S3:** Number of observed children by population, age, and sex.
**Table S4:** Data collection.
**Table S5:** Parameter estimates for the models of maternal child‐holding rates.
**Table S6:** Parameter estimates for models of maternal breastfeeding rates.
**Table S7:** Parameter estimates for models of child‐holding rates by allomothers, including older children, adolescents, and adults.
**Table S8:** Parameter estimates for models of child‐holding rates by young allomaternal caregivers, including older children and adolescents.
**Table S9:** Parameter estimates for models of focal children's participation rates in mixed‐age peer groups.

## Data Availability

All relevant data and code for reproducing the analyses and figures are available at the following Figshare records: https://doi.org/10.6084/m9.figshare.28303322 and https://doi.org/10.6084/m9.figshare.28304084 (Tanaka et al. 2025a, 2025b).

## References

[ajpa70345-bib-0001] Bowlby, J. 1969. Attachment and Loss. Vol. 1, Attachment. Basic Books.

[ajpa70345-bib-0002] Chaudhary, N. , A. Page , G. D. Salali , et al. 2024. “Hunter‐Gatherer Children's Close‐Proximity Networks: Similarities and Differences With Cooperative and Communal Breeding Systems.” Evolutionary Human Sciences 6: e11. 10.1017/ehs.2024.1. 38516373 PMC10955362

[ajpa70345-bib-0003] Chaudhary, N. , G. D. Salali , and A. Swanepoel . 2024. “Sensitive Responsiveness and Multiple Caregiving Networks Among Mbendjele BaYaka Hunter‐Gatherers: Potential Implications for Psychological Development and Well‐Being.” Developmental Psychology 60, no. 3: 422–440. 10.1037/dev0001601. 37956035

[ajpa70345-bib-0004] Draper, P. 1976. “Social and Economic Constraints on Child Life Among the !Kung.” In Kalahari Hunter‐Gatherers: Studies of the !Kung San and Their Neighbors, edited by R. B. Lee and I. DeVore , 199–217. Harvard University Press.

[ajpa70345-bib-0005] Emmott, E. , and A. Page . 2019. “Alloparenting.” In Encyclopedia of Evolutionary Psychological Science, edited by T. K. Shackelford and V. A. Weekes‐Shackelford , 1–14. Springer International Publishing. 10.1007/978-3-319-16999-6_2253-1.

[ajpa70345-bib-0006] Gallois, S. , M. J. Lubbers , B. Hewlett , and V. Reyes‐García . 2018. “Social Networks and Knowledge Transmission Strategies Among Baka Children, Southeastern Cameroon.” Human Nature 29, no. 4: 442–463. 10.1007/s12110-018-9328-0. 30357606 PMC6208833

[ajpa70345-bib-0007] Hawkes, K. , J. F. O'Connell , N. G. B. Jones , H. Alvarez , and E. L. Charnov . 1998. “Grandmothering, Menopause, and the Evolution of Human Life Histories.” Proceedings of the National Academy of Sciences of the United States of America 95, no. 3: 1336–1339. 10.1073/pnas.95.3.1336. 9448332 PMC18762

[ajpa70345-bib-0008] Helfrecht, C. , J. W. Roulette , A. Lane , B. Sintayehu , and C. L. Meehan . 2020. “Life History and Socioecology of Infancy.” American Journal of Physical Anthropology 173, no. 4: 619–629. 10.1002/ajpa.24145. 32955732

[ajpa70345-bib-0009] Hewlett, B. S. 1991. Intimate Fathers: The Nature and Context of Aka Pygmy Paternal Infant Care, edited by S. Hewlett Barry . University of Michigan Press.

[ajpa70345-bib-0010] Hewlett, B. S. , and S. Winn . 2014. “Allomaternal Nursing in Humans.” Current Anthropology 55, no. 2: 200–229. 10.1086/675657. 24991682

[ajpa70345-bib-0011] Hirasawa, A. 2005. “Infant Care Among the Sedentarized Baka Hunter‐Gatherers in Southeastern Cameroon.” In Hunter‐Gatherer Childhoods: Evolutionary, Developmental & Cultural Perspectives, edited by B. S. Hewlett and M. E. Lamb , 365–384. Aldine Transaction.

[ajpa70345-bib-0012] Hongo, S. , Y. Nakashima , E. F. Akomo‐Okoue , and F. L. Mindonga‐Nguelet . 2022. “Seasonality in Daily Movement Patterns of Mandrills Revealed by Combining Direct Tracking and Camera Traps.” Journal of Mammalogy 103, no. 1: 159–168. 10.1093/jmammal/gyab141. 35087330 PMC8789762

[ajpa70345-bib-0013] Hrdy, S. B. 1999. Mother Nature: A History of Mothers, Infants, and Natural Selection. Pantheon. 10.1006/anbe.1999.1378 10792945

[ajpa70345-bib-0014] Hrdy, S. B. 2009. Mothers and Others: The Evolutionary Origins of Mutual Understanding. Harvard University Press.

[ajpa70345-bib-0015] Jang, H. , K. R. Janmaat , V. Kandza , and A. H. Boyette . 2022. “Girls in Early Childhood Increase Food Returns of Nursing Women During Subsistence Activities of the BaYaka in the Republic of Congo.” Proceedings of the Royal Society B: Biological Sciences 289, no. 1987: 20221407. 10.1098/rspb.2022.1407. PMC9667358 36382518

[ajpa70345-bib-0016] Konner, M. 1977. “Infancy Among the Kalahari Desert San.” In Culture and Infancy: Variations in the Human Experience, edited by H. Liederman , S. Tulkin , and A. Rosenfeld , 287–328. Academic Press.

[ajpa70345-bib-0017] Konner, M. 2017. “Hunter‐Gatherer Infancy and Childhood: The !Kung and Others.” In Hunter‐Gatherer Childhoods, edited by B. S. Hewlett and M. E. Lamb , 19–64. Routledge.

[ajpa70345-bib-0018] Kramer, K. L. 2005. “Children's Help and the Pace of Reproduction: Cooperative Breeding in Humans.” Evolutionary Anthropology: Issues, News, and Reviews 14, no. 6: 224–237. 10.1002/evan.20082.

[ajpa70345-bib-0019] Kramer, K. L. 2010. “Cooperative Breeding and Its Significance to the Demographic Success of Humans.” Annual Review of Anthropology 39: 417–436. 10.1146/annurev.anthro.012809.105054.

[ajpa70345-bib-0020] Kruger, A. C. , and M. Konner . 2010. “Who Responds to Crying?: Maternal Care and Allocare Among the !Kung.” Human Nature 21, no. 3: 309–329. 10.1007/s12110-010-9095-z.

[ajpa70345-bib-0021] Lew‐Levy, S. , and D. Amir . 2026. “Children as Agents of Cultural Adaptation.” Behavioral and Brain Sciences 49: e225. 10.1017/S0140525X24001377. 39721974

[ajpa70345-bib-0022] Meehan, C. L. , and S. Hawks . 2013. “Cooperative Breeding and Attachment Among the Aka Foragers.” In Attachment Reconsidered: Cultural Perspectives on a Western Theory, edited by N. Quinn and J. M. Mageo , 85–113. Palgrave Macmillan US.

[ajpa70345-bib-0023] Meehan, C. L. , C. Helfrecht , and R. J. Quinlan . 2014. “Cooperative Breeding and Aka Children's Nutritional Status: Is Flexibility Key?” American Journal of Physical Anthropology 153, no. 4: 513–525. 10.1002/ajpa.22415. 24452414

[ajpa70345-bib-0024] Page, A. , E. H. Emmott , M. Dyble , et al. 2021. “Children Are Important Too: Juvenile Playgroups and Maternal Childcare in a Foraging Population, the Agta.” Philosophical Transactions of the Royal Society, B: Biological Sciences 376, no. 1827: 20200026. 10.1098/rstb.2020.0026. PMC8090817 33938270

[ajpa70345-bib-0025] Page, A. , A. B. Migliano , M. Dyble , D. Major‐Smith , S. Viguier , and A. Hassan . 2023. “Sedentarization and Maternal Childcare Networks: Role of Risk, Gender and Demography.” Philosophical Transactions of the Royal Society, B: Biological Sciences 378, no. 1868: 20210435. 10.1098/rstb.2021.0435. PMC9703224 36440566

[ajpa70345-bib-0026] Posit Team . 2023. RStudio: Integrated Development Environment for R. 2023.09.1+494. Posit Software, PBC. http://www.posit.co/.

[ajpa70345-bib-0027] R Core Team . 2025. R: A Language and Environment for Statistical Computing. 4.5.0. R Foundation for Statistical Computing. https://www.r‐project.org/.

[ajpa70345-bib-0028] Takada, A. 2010. “Changes in Developmental Trends of Caregiver–Child Interactions Among the San: Evidence From the !Xun of Northern Namibia.” African Study Monographs, Supplementary Issue 40: 155–177. 10.14989/96291.

[ajpa70345-bib-0029] Takada, A. 2014. “Kinship and Caregiving Practices Among the Ekoka !Xun.” In Southern African Khoisan Kinship Systems. Research in Khoisan Studies, edited by A. Barnard and G. Boden , 99–120. Rüdiger Köppe Verlag.

[ajpa70345-bib-0030] Takada, A. 2020. The Ecology of Playful Childhood: The Diversity and Resilience of Caregiver–Child Interactions Among the San of Southern Africa. Springer International Publishing.

[ajpa70345-bib-0031] Takada, A. 2022. Hunters Among Farmers: The !Xun of Ekoka. University of Namibia Press.

[ajpa70345-bib-0032] Tanaka, A. , H. Jang , S. Hongo , and A. Takada . 2025a. “Dataset for ‘Reframing Mother–Child Closeness and Cooperative Caregiving in Hunter‐Gatherer Societies’”. 10.6084/m9.figshare.28303322.42601348

[ajpa70345-bib-0033] Tanaka, A. , H. Jang , S. Hongo , and A. Takada . 2025b. “R Code for ‘Reframing Mother–Child Closeness and Cooperative Caregiving in Hunter‐Gatherer Societies’”. 10.6084/m9.figshare.28304084.42601348

[ajpa70345-bib-0034] Tronick, E. Z. , G. A. Morelli , and S. Winn . 1987. “Multiple Caretaking of Efe (Pygmy) Infants.” American Anthropologist 89, no. 1: 96–106. 10.1525/aa.1987.89.1.02a00050.

[ajpa70345-bib-0035] Visine, A. E. S. , A. H. Boyette , Y. R. Ouamba , S. Lew‐Levy , M. S. Sarma , and H. Jang . 2024. “BaYaka Mothers Balance Childcare and Subsistence Tasks During Collaborative Foraging in Congo Basin.” Scientific Reports 14, no. 1: 24893. 10.1038/s41598-024-75467-1. 39438509 PMC11496537

[ajpa70345-bib-0036] Wood, S. , and F. Scheipl . 2025. gamm4: Generalized Additive Mixed Models Using 'mgcv' and 'lme4'. R Package Version 0.2‐7. https://CRAN.R‐project.org/package=gamm4.

