# Supplementary material for: Reframing Mother–Child Closeness and Cooperative Caregiving: A Comparative Study of Two Hunter‐Gatherer Populations
Source: Am J Biol Anthropol. 2026 Aug 14;190(4):e70345. doi: 10.1002/ajpa.70345 (PMC13476358; doi:10.1002/ajpa.70345)
Supplement: Supplementary file 1 [file AJPA-190-e70345-s001.docx]

**American Journal of Biological Anthropology**

**Supporting Information for**

**Reframing Mother–Child Closeness and Cooperative Caregiving: A Comparative Study of Two Hunter-Gatherer Populations**

Ayana Tanaka, Haneul Jang, Shun Hongo, Akira Takada

**Email:** tanaka.ayana.y55@kyoto-u.jp

**This PDF file includes:**

Tables S1 to S9

**Ethnographic Description**

**!Xun**: Southern African hunter-gatherers, collectively known as the San, inhabit the Kalahari Desert, which extends across several countries. This study focused on the !Xun, one of the San groups, who live in Ekoka village, located 25 km southeast of Okongo in the Ohangwena Region of north-central Namibia. The !Xun are closely related to but distinct from the !Kung (or Juǀʼhoan), differing in language and geographic distribution. Ekoka was established by missionaries from the Evangelical Lutheran Ovambo/Kavango Church (ELOC). In 1998, it had a population of 281 San, most of whom were !Xun or ǂAkhoe (another San group).

The !Xun live in huts, each typically occupied by a nuclear family; elderly family members sometimes reside with their children.

Hunting is conducted with bows and arrows, guns, traps, and dogs. Meat obtained through hunting is customarily shared with the owners of borrowed hunting tools. At the time of the survey, however, only some !Xun engaged in hunting.

The livelihoods of the Ekoka !Xun were based primarily on farming and livestock herding at the time of the focal observations. Their main farming activity is the cultivation of *mahangu* (pearl millet), which is managed collectively on government-administered farmland. In typical years, the government supplies grain seeds, while harvested crops are distributed during periods of drought. Opportunities to earn cash are limited, consisting mainly of government pension payments and occasional work for agro-pastoralists who have settled nearby.

Public facilities, including a school and a clinic, are located in Ekoka. At the time of the focal observations, most children were not attending school. Although a kindergarten had been established in the village, classroom activities during the focal observations were observed only among three-year-old children and accounted for just 3% of the total observation time (Takada, 2010, 2022).

**Baka:** Central African hunter-gatherers inhabit the rainforests of the Congo Basin, which extend across several countries. This study focused on the Baka, one of these hunter-gatherer groups, who live in the Lanjo settlement of Ngato Ancien village in the Yokadouma District of southeastern Cameroon. The Baka are the only hunter-gatherer group living in Lanjo. They lead a semi-sedentary, semi-nomadic lifestyle, moving among roadside settlements, farming camps, and forest camps. During the focal observations, the Baka in Lanjo stayed overnight in the village and at forest camps at an approximate ratio of 2:1 (Tanaka, unpublished survey).

Baka dwellings comprise three main types: wooden houses, mud houses, and *móngulu*, dome-shaped huts with leaves inserted into a wooden frame. At forest camps, each *móngulu* is typically occupied by a nuclear family, and several such huts form a camp.

Hunting is conducted primarily with snares and sometimes with guns. Meat obtained through hunting is commonly shared among group members (Sekino, Otsuka, and Yasuoka, 2023).

The livelihoods of the Baka were based primarily on farming, hunting, gathering, and exchange with neighboring farmers at the time of the focal observations. Although the Baka do not have fields as large as those of neighboring farmers, they have their own fields along the main roads. Plantains and cassava are the main staple foods in the village and farming camps, whereas forest-camp diets rely on wild yams and wild animal protein (Yasuoka, 2006). The Baka also provide labor and forest resources to neighboring farmers in exchange for money and goods.

There are no public facilities in Lanjo. The primary school serving children from Lanjo is located in another settlement and had been closed since 2019 because of a lack of teachers. At the time of the focal observations, the children were therefore not attending school.

Table S1. Age-sex composition of !Xun residents of Ekoka (census by Takada in 1998). The !Xun focal-observation data were collected in 2004, whereas the demographic data presented for reference are from the 1998 census.

| !Xun | male | female |
| --- | --- | --- |
| 0－4 yrs. | 5 | 10 |
| 5－14 yrs. | 23 | 16 |
| 15－24 yrs. | 6 | 11 |
| 25－34 yrs. | 9 | 8 |
| 35－44 yrs. | 12 | 6 |
| 45 yrs. － | 13 | 15 |
| Total | 68 | 66 |

Table S2. Age-sex composition of Baka residents in Lanjo (census by Tanaka in 2023). Individuals with a Bantu farmer father and a Baka hunter-gatherer mother were excluded from the Baka demographic counts. Children aged 0–4 years with this parentage were also excluded from the focal observations.

| Baka | male | female |
| --- | --- | --- |
| 0－4 yrs. | 13 | 10 |
| 5－14 yrs. | 15 | 22 |
| 15－24 yrs. | 14 | 16 |
| 25－34 yrs. | 12 | 12 |
| 35－44 yrs. | 6 | 7 |
| 45 yrs. － | 6 | 5 |
| Total | 66 | 72 |

Table S3. Number of observed children by population, age, and sex

| Population | 1-11 months | | 12-23 months | | 24-35 months | | 36-47 months | | 48-59 months | | Total |
| --- | --- | --- | --- | --- | --- | --- | --- | --- | --- | --- | --- |
|  | Boy | Girl | Boy | Girl | Boy | Girl | Boy | Girl | Boy | Girl |  |
| Baka | 3 | 1 | 3 | 1 | 1 | 4 | 3 | 2 | 4 | 1 | 23 |
| !Xun | 3 | 0 | 2 | 2 | 1 | 2 | 3 | 0 | 2 | 2 | 17 |

Table S4. Data collection.

| Population | Observation days per child | Observation time per child | Number of 30-s intervals per child |
| --- | --- | --- | --- |
| Baka | 2 days | 16 hours | 1,920 |
| !Xun | 1 day | 8 hours | 960 |

Table S5. Parameter estimates for the models of maternal child-holding rates.

| **Model 1 (Age-only model): Selected model** | |
| --- | --- |
| **Model structure** | α + f(A) + rID1 + rID2 |
| **Fixed effect** |  |
| **Parametric term [estimate (95% CI)]** |  |
| Intercept [α] | −4.44 (−5.01 to −3.87) |
| **Smooth term [edf]** |  |
| Age [f(A)] | 1.00 |
| **Random effect (standard deviation)** |  |
| Focal child [rID1] | 1.39 |
| Observation [rID2] | 1.77 |
| **Model 2 (Age + population model)** | |
| **Model structure** | α + β_P + f(A) + rID1 + rID2 |
| **Fixed effect** |  |
| **Parametric term [estimate (95% CI)]** |  |
| Intercept [α] | −4.88 (−5.81 to −3.94) |
| Population (Baka vs !Xun) [β_P] | 0.69 (−0.45 to 1.83) |
| **Smooth term [edf]** |  |
| Age [f(A)] | 1.00 |
| **Random effect (standard deviation)** |  |
| Focal child [rID1] | 1.35 |
| Observation [rID2] | 1.78 |
| **Model 3 (Age × population model)** | |
| **Model structure** | α + β_P + f(A) × P + rID1 + rID2 |
| **Fixed effect** |  |
| **Parametric term [estimate (95% CI)]** |  |
| Intercept [α] | −4.87 (−5.84 to −3.90) |
| Population (Baka vs !Xun) [β_P] | 0.68 (−0.51 to 1.87) |
| **Smooth term [edf]** |  |
| Age × population (Baka) [f(A) × P] | 1.00 |
| Age × population (!Xun) [f(A) × P] | 1.00 |
| **Random effect (standard deviation)** |  |
| Focal child [rID1] | 1.35 |
| Observation [rID2] | 1.78 |

Table S6. Parameter estimates for models of maternal breastfeeding rates.

| **Model 1 (Age-only model)** | |
| --- | --- |
| **Model structure** | α + f(A) + rID1 + rID2 |
| **Fixed effect** |  |
| **Parametric term [estimate (95% CI)]** |  |
| Intercept [α] | −7.51 (−9.38 to −5.63) |
| **Smooth term [edf]** |  |
| Age [f(A)] | 1.79 |
| **Random effect (standard deviation)** |  |
| Focal child [rID1] | 1.86 |
| Observation [rID2] | 0.96 |
| **Model 2 (Age + population model): Selected model** | |
| **Model structure** | α + β_P + f(A) + rID1 + rID2 |
| **Fixed effect** |  |
| **Parametric term [estimate (95% CI)]** |  |
| Intercept [α] | −8.79 (−11.13 to −6.46) |
| Population (Baka vs !Xun) [β_P] | 1.69 (0.28 to 3.09) |
| **Smooth term [edf]** |  |
| Age [f(A)] | 2.16 |
| **Random effect (standard deviation)** |  |
| Focal child [rID1] | 1.56 |
| Observation [rID2] | 0.96 |
| **Model 3 (Age × population model)** | |
| **Model structure** | α + β_P + f(A) × P + rID1 + rID2 |
| **Fixed effect** |  |
| **Parametric term [estimate (95% CI)]** |  |
| Intercept [α] | −7.47 (−9.24 to −5.69) |
| Population (Baka vs !Xun) [β_P] | 0.37 (−2.43 to 3.17) |
| **Smooth term [edf]** |  |
| Age × population (Baka) [f(A) × P] | 2.19 |
| Age × population (!Xun) [f(A) × P] | 1.00 |
| **Random effect (standard deviation)** |  |
| Focal child [rID1] | 1.47 |
| Observation [rID2] | 0.96 |

Table S7. Parameter estimates for models of child-holding rates by allomothers, including older children, adolescents, and adults.

| **Model 1 (Age-only model)** | |
| --- | --- |
| **Model structure** | α + f(A) + rID1 + rID2 |
| **Fixed effect** |  |
| **Parametric term [estimate (95% CI)]** |  |
| Intercept [α] | −3.25 (−3.62 to −2.89) |
| **Smooth term [edf]** |  |
| Age [f(A)] | 1.00 |
| **Random effect (standard deviation)** |  |
| Focal child [rID1] | 0.90 |
| Observation [rID2] | 1.47 |
| **Model 2 (Age + population model): Selected model** | |
| **Model structure** | α + β_P + f(A) + rID1 + rID2 |
| **Fixed effect** |  |
| **Parametric term [estimate (95% CI)]** |  |
| Intercept [α] | −3.80 (−4.41 to −3.20) |
| Population (Baka vs !Xun) [β_P] | 0.84 (0.10 to 1.58) |
| **Smooth term [edf]** |  |
| Age [f(A)] | 1.00 |
| **Random effect (standard deviation)** |  |
| Focal child [rID1] | 0.84 |
| Observation [rID2] | 1.46 |
| **Model 3 (Age × population model)** | |
| **Model structure** | α + β_P + f(A) × P + rID1 + rID2 |
| **Fixed effect** |  |
| **Parametric term [estimate (95% CI)]** |  |
| Intercept [α] | −3.99 (−4.63 to −3.35) |
| Population (Baka vs !Xun) [β_P] | 1.04 (0.28 to 1.80) |
| **Smooth term [edf]** |  |
| Age × population (Baka) [f(A) × P] | 1.00 |
| Age × population (!Xun) [f(A) × P] | 1.85 |
| **Random effect (standard deviation)** |  |
| Focal child [rID1] | 0.78 |
| Observation [rID2] | 1.47 |

Table S8. Parameter estimates for models of child-holding rates by young allomaternal caregivers, including older children and adolescents.

| **Model 1 (Age-only model)** | |
| --- | --- |
| **Model structure** | α + f(A) + rID1 + rID2 |
| **Fixed effect** |  |
| **Parametric term [estimate (95% CI)]** |  |
| Intercept [α] | −3.93 (−4.35 to −3.52) |
| **Smooth term [edf]** |  |
| Age [f(A)] | 1.00 |
| **Random effect (standard deviation)** |  |
| Focal child [rID1] | 1.04 |
| Observation [rID2] | 1.60 |
| **Model 2 (Age + population model)** | |
| **Model structure** | α + β_P + f(A) + rID1 + rID2 |
| **Fixed effect** |  |
| **Parametric term [estimate (95% CI)]** |  |
| Intercept [α] | −4.32 (−5.03 to −3.60) |
| Population (Baka vs !Xun) [β_P] | 0.58 (−0.31 to 1.46) |
| **Smooth term [edf]** |  |
| Age [f(A)] | 1.00 |
| **Random effect (standard deviation)** |  |
| Focal child [rID1] | 1.03 |
| Observation [rID2] | 1.60 |
| **Model 3 (Age × population model): Selected model** | |
| **Model structure** | α + β_P + f(A) × P + rID1 + rID2 |
| **Fixed effect** |  |
| **Parametric term [estimate (95% CI)]** |  |
| Intercept [α] | −4.78 (−5.72 to −3.85) |
| Population (Baka vs !Xun) [β_P] | 1.06 (0.02 to 2.11) |
| **Smooth term [edf]** |  |
| Age × population (Baka) [f(A) × P] | 1.00 |
| Age × population (!Xun) [f(A) × P] | 2.08 |
| **Random effect (standard deviation)** |  |
| Focal child [rID1] | 0.89 |
| Observation [rID2] | 1.60 |

Table S9. Parameter estimates for models of focal children’s participation rates in mixed-age peer groups.

| **Model 1 (Age-only model): Selected model** | |
| --- | --- |
| **Model structure** | α + f(A) + rID1 + rID2 |
| **Fixed effect** |  |
| **Parametric term [estimate (95% CI)]** |  |
| Intercept [α] | −2.75 (−3.49 to −2.02) |
| **Smooth term [edf]** |  |
| Age [f(A)] | 3.00 |
| **Random effect (standard deviation)** |  |
| Focal child [rID1] | 1.98 |
| Observation [rID2] | 1.73 |
| **Model 2 (Age + population model)** | |
| **Model structure** | α + β_P + f(A) + rID1 + rID2 |
| **Fixed effect** |  |
| **Parametric term [estimate (95% CI)]** |  |
| Intercept [α] | −2.97 (−4.12 to −1.81) |
| Population (Baka vs !Xun) [β_P] | 0.34 (−1.11 to 1.79) |
| **Smooth term [edf]** |  |
| Age [f(A)] | 3.05 |
| **Random effect (standard deviation)** |  |
| Focal child [rID1] | 1.98 |
| Observation [rID2] | 1.73 |
| **Model 3 (Age × population model)** | |
| **Model structure** | α + β_P + f(A) × P + rID1 + rID2 |
| **Fixed effect** |  |
| **Parametric term [estimate (95% CI)]** |  |
| Intercept [α] | −2.67 (−3.79 to −1.56) |
| Population (Baka vs !Xun) [β_P] | 0.08 (−1.36 to 1.53) |
| **Smooth term [edf]** |  |
| Age × population (Baka) [f(A) × P] | 3.10 |
| Age × population (!Xun) [f(A) × P] | 1.00 |
| **Random effect (standard deviation)** |  |
| Focal child [rID1] | 1.96 |
| Observation [rID2] | 1.73 |

**References**

Sekino, A., R. Otsuka, and H. Yasuoka. 2023. "Haphazard Sharing of Plant Food among the Baka Hunter-Gatherers in Southeast Cameroon." *African Study Monographs,* Supplementary Issue 62: 81–103. <https://doi.org/10.14989/286829>.

Takada, A. 2010. "Changes in Developmental Trends of Caregiver–Child Interactions Among the San: Evidence From the !Xun of Northern Namibia." *African Study Monographs,* Supplementary Issue 40: 155–177. <https://doi.org/10.14989/96291>.

Takada, A. 2022. *Hunters Among Farmers: The !Xun of Ekoka*. Windhoek: University of Namibia Press.

Yasuoka, H. 2006. "Long-Term Foraging Expeditions (Molongo) Among the Baka Hunter-Gatherers in the Northwestern Congo Basin, With Special Reference to the “Wild Yam Question”." *Human Ecology* 34: 275–296. <https://doi.org/10.1007/s10745-006-9017-1>.
